# Follistatin expressed in mechanically-damaged salivary glands of male mice induces proliferation of CD49f^+^ cells

**DOI:** 10.1038/s41598-020-77004-2

**Published:** 2020-11-17

**Authors:** A. Ikeda, T. Yamamoto, J. Mineshiba, S. Takashiba

**Affiliations:** 1 Department of Periodontics and Endodontics, Okayama University Hospital, 2-5-1 Shikata-cho, Kita-ku, Okayama, 700-8525 Japan; 2 Department of Pathophysiology - Periodontal Science, Okayama University Graduate School of Medicine, Dentistry and Pharmaceutical Sciences, 2-5-1 Shikata-cho, Kita-ku, Okayama, 700-8525 Japan; 3 Hanamizuki Dental Clinic, 285-2 Hirano, Kita-ku, Okayama, 701-0151 Japan

**Keywords:** Adult stem cells, Cell biology

## Abstract

Salivary glands (SGs) are very important for maintaining the physiological functions of the mouth. When SGs regenerate and repair from various damages, including mechanical, radiological, and immune diseases, acinar and granular duct cells originate from intercalated duct cells. However, the recovery is often insufficient because of SGs' limited self-repair function. Furthermore, the precise repair mechanism has been unclear. Here, we focused on CD49f, one of the putative stem cell markers, and characterized CD49f positive cells (CD49f^+^ cells) isolated from male murine SGs. CD49f^+^ cells possess self-renewal ability and express epithelial and pluripotent markers. Compared to CD49f negative cells, freshly isolated CD49f^+^ cells highly expressed inhibin beta A and beta B, which are components of activin that has anti-proliferative effects. Notably, an inhibitor of activin, follistatin was expressed in mechanically-damaged SGs, meanwhile no follistatin was expressed in normal SGs in vivo. Moreover, sub-cultured CD49f^+^ cells highly expressed both *Follistatin* and a series of proliferative genes, expressions of which were decreased by *Follistatin* siRNA. These findings indicated that the molecular interaction between activin and follistatin may induce CD49f^+^ cells proliferation in the regeneration and repair of mouse SGs.

## Introduction

Saliva has many abilities, including the protection of mucous membranes, buffer capacity, digestion, and antibacterial activity^1^. Through these effects, saliva can contribute to prevent oral infectious diseases, such as dental caries^2^ and periodontitis^3^, and to promote wound healing^4^. Therefore, salivary glands, the saliva secreting organ, is crucial for oral health. Salivary glands consist of many kinds of cells and secrete many kinds of proteins, such as amylase, kallikrein^5^, nerve growth factor, and epidermal growth factor^6^. Since salivary glands have a complex structure with various kinds of cells, the molecular mechanisms for embryonic development and postnatal remodeling remain unclear. Consequently, once they are damaged by Sjögren’s syndrome or radiation therapy for head or neck cancer, they cannot completely recover their abilities because they have a limited self-repair function^7^. This is the reason why we do not have effective means for their regeneration, so far.

In general, during the regeneration and repair of mature organs, it is important to control the appropriate number and kinds of cells for the proper recovery of organ size and structure. To achieve these goals, there are two possibilities of cell replacement mechanisms: one is proliferation and differentiation from stem or precursor cell pools, and the other is self-duplication of existing mature cells^8^. Generally, it is believed that somatic stem cells are involved in the turnover and repair of mature tissues. As represented by hematopoietic stem cells^9^, keratinocyte stem cells^10,11^, and mesenchymal stem cells in bone marrow^12^, somatic stem cells are present in various organs, and have a limited potency to differentiate into mature cells only in the specific organ where they exist as one of the components. In salivary glands, although there is still room for consideration of the stem cells location, one of the hypotheses is that somatic stem cells may exist in intercalated ducts because it is known that acinar and granular duct cells originate from intercalated duct cells during remodeling^13–15^. However, somatic stem cells in salivary glands have not been specifically defined, because only few specific markers have been found as stem cells in salivary glands^16,17^.

A differentiation marker, CD49f, also known as integrin subunit α6, is used as the marker for keratinocyte stem cells^10^ and breast cancer stem cells^18^. Meanwhile, it is used as the putative marker for salivary stem cells^19,20^. The general molecular structure of CD49f allows its heterodimerization with integrin β1 chain (CD29) to form very late activation antigen 6 (VLA-6) and with integrin β4 chain (CD104) to form tumor surface protein 180 (TSP-180). The heterodimers can bind laminin, which is one of the major epithelial extracellular matrix proteins. Since CD49f is a putative marker for salivary stem cells^19,20^, it promotes submandibular glands morphogenesis and differentiation^21^. CD49f was expressed by mouse endothelial and epithelial cells on embryonic day 13, and branching morphogenesis of mouse submandibular glands were perturbed by an anti-CD49f monoclonal antibody in the organ culture^22^. CD49f was also observed in epithelial cells that compose the excretory duct of mouse submandibular glands^23^. Furthermore, CD49f positive cells (CD49f^+^ cells) isolated from mouse submandibular glands differentiated into the hepatic and pancreatic lineages^24^. However, the underlying mechanisms for the morphogenesis and repair/regeneration by CD49f^+^ cell populations still remain unclear.

It is well known that submandibular glands of male rodents have the specific organ, Granular convoluted tubules (GCT), and GCT secret many kinds of growth factors and bioactive substances^25^. This character of GCT could be beneficial for the isolation of growth factors and their related factors required for the repair of salivary gland.

In the present study, we hypothesized that CD49f^+^ cells are related to repair/regeneration in male mouse salivary glands as the fundamental research question. To reveal how CD49f^+^ cells are related to repair/regeneration in male mouse salivary glands, we confirmed whether CD49f^+^ cells in male mouse salivary glands have multi-potency as somatic stem cells firstly. Moreover, to identify the molecular functions regulated by CD49f in male mouse salivary glands, we investigated what kind of growth factors are highly expressed by CD49f^+^ cells isolated from male mouse salivary glands, compared to CD49f negative cells (CD49f^-^ cells) of male mouse salivary glands, and how these growth factors are related to cell proliferation in regeneration and repair of salivary glands.

## Results

### Ratio of CD49f^+^ cells and growth factor mRNA expression

**Figure 1:**
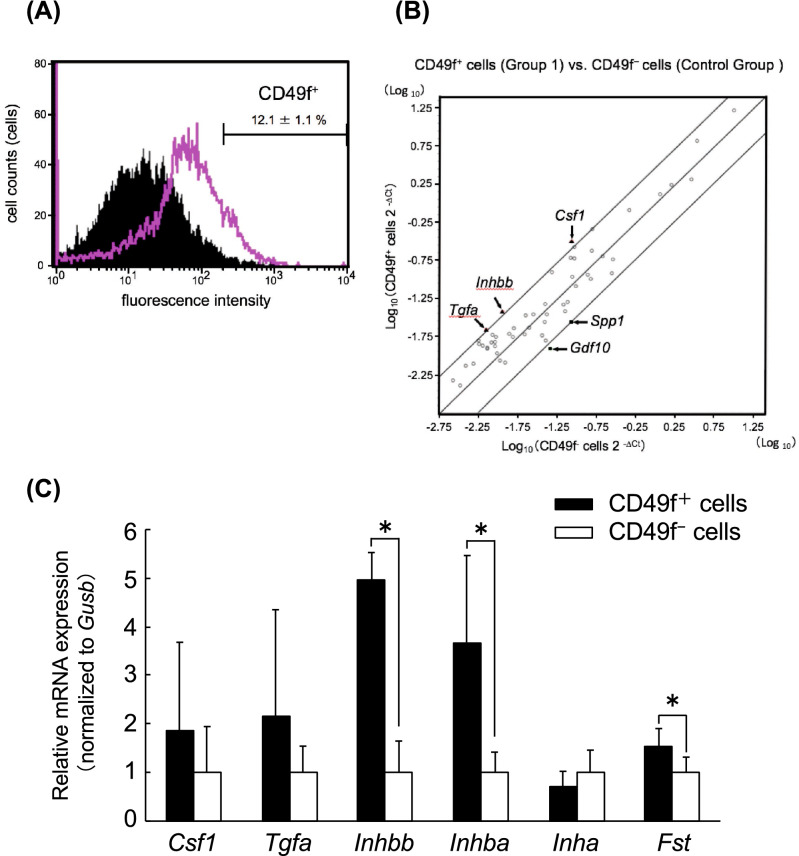
(**A**) Flow-cytometric analysis of the CD49f^+^ cell fraction of freshly isolated cells from salivary glands of 5-week-old male mice. The pink-line area shows how the cells reacted to the CD49f antibody, while the black area is the negative control group with the isotype control antibody. One set of experiments was performed using the pooled cells from salivary glands of 3 mice, and the value represents the ratio of CD49f^+^ fractions from 3 sets of experiments. A typical image is shown. (**B**) Overview of scatter plot on 84 spotted genes in the Mouse Growth Factors RT^2^ Profiler PCR Array by plotting log_10_ transformed 2^-ΔCt^ values between freshly-isolated CD49f^+^ cells (*y*-axis) and CD49f^-^ cells (*x*-axis). The central line indicates an unchanged gene expression, and the boundaries represent a threefold cut-off difference. Triangle dots represent upregulated genes, and square dots represent downregulated genes in CD49f^+^ cells, compared to CD49f^-^ cells. The gene symbols are indicated as follows: *Colony stimulating factor 1* (*Csf1*), *Inhibin beta b* (*Inhbb*), *Transforming growth factor alpha* (*Tgfa*), S*ecreted phosphoprotein 1* (*Spp1*), and *Growth differentiation factor 10* (*Gdf10*). (**C**) RT-qPCR analysis for up-regulated genes is shown in (**B**) for freshly-isolated CD49f cells. mRNA quantities of *Csf1*, *Tgfa*, *Inhbb*, *Inhba*, *Inha*, and *Follistatin (Fst)* were determined relative to *Glucuronidase beta* (*Gusb*) by using the ΔΔCt method, and fold induction is shown. CD49f^+^ cells (solid bars), CD49f^-^ cells (open bars). One set of experiments was performed using the total RNA extracted from CD49f^+^ and CD49f^-^ cells fractionated from the salivary glands of 3 mice, and 3 sets of experiments carried out independently. *P < 0.05, Student’s *t* test.

Flow cytometry analysis indicated that the ratio of freshly isolated CD49f^+^ fractions from salivary glands of five-week-old male C57BL/6 mice was 12.1 ± 1.1% (Fig. 1A). To characterize CD49f^+^ cells, gene expression profiling was performed between freshly-isolated CD49f^+^ and CD49f^-^ cells using a PCR array. Among 84 spotted genes, we selected genes with relatively high mRNA accumulation levels (threshold cycle (Ct) < 30) and 5 genes in the CD49f^+^ fractions had a threefold higher expression level than CD49f^-^ fractions (Fig. 1B). The expression patterns were verified by using real-time RT-PCR (RT-qPCR) between CD49f^+^ and CD49f^-^ fractions, and there was a statistically significant difference in the expression of 3 genes. *Inhibin beta b* (*Inhbb*) expression increased by approximately fivefold in CD49f^+^ cells (Fig. 1C), conversely, *Secreted phosphoprotein 1* (*Spp1*) and *Growth differentiation factor* 10 (*Gdf10*) showed a decrease greater than tenfold in CD49f^+^ (Supplementary Figure 1) fraction.

### mRNA expression of growth factors related to activins and inhibins

**Figure 2:**
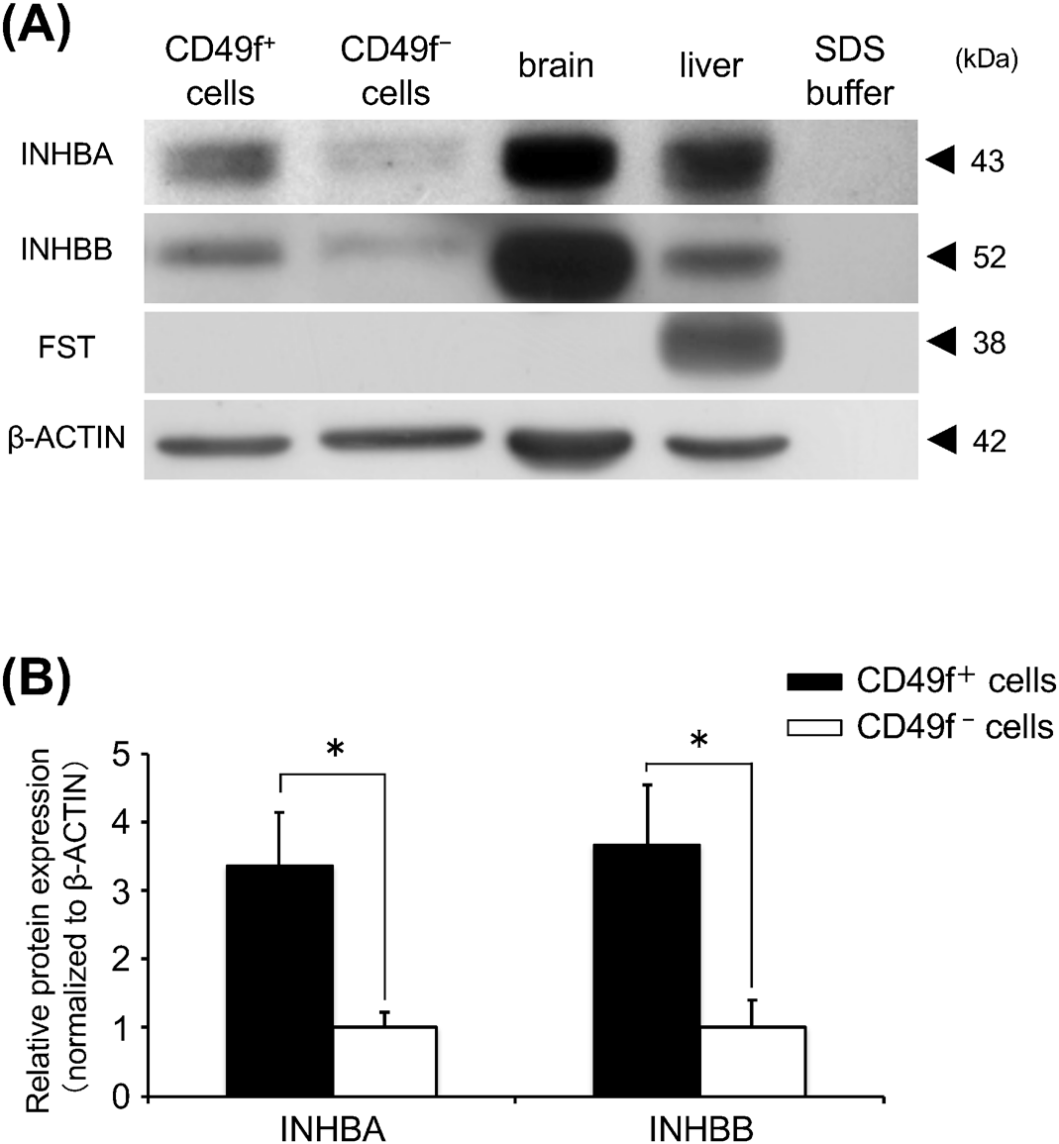
(**A**) Western blot analysis for freshly-isolated cells: Proteins of INHBA, INHBB, and follistatin (FST) were detected from protein extracts (30 μg/lane). β-actin was used as an internal control by re-probing. Brain protein extracts, positive control of INHBA and INHBB; liver protein extracts, positive control of FST; SDS buffer, negative control. Typical blotting images are shown from 3 independent experiments. One set of experiments was performed using protein extracts of CD49f^+^ and CD49f^-^ cells fractionated from the salivary glands of 3 mice, and 3 sets of experiments carried out independently. (**B**) Densitometric analysis for protein levels of INHBA, INHBB, and FST. Signals were normalized to β-actin. One set of experiments was performed using protein extracts of CD49f^+^ and CD49f^-^ cells fractionated from the salivary glands of 3 mice, and 3 sets of experiments carried out independently. *P < 0.05, Student’s *t* test.

*Inhbb* mRNA expression was validated by using RT-qPCR (Fig. 1C; *Inhbb*). In addition, mRNA expression analysis of 3 inhbb-related genes, *Inha*, *Inhba*, and *Follistatin,* revealed that *Inhba* and *Follistatin* expressions increased along with *Inhbb* in freshly isolated CD49f^+^, compared to CD49f^–^ (each 3.7, 5.0, and 1.5 -fold, respectively, *P* < 0.05), but *Inha* expression presented a similar level for both fractions (Fig. 1C). Regarding the protein level, INHBA and INHBB expression in freshly isolated CD49f^+^ fractions were significantly high, compared to CD49f^-^ fractions (3.4 and 3.7 -fold, respectively, *P* < 0.05), but follistatin presented an undetectable level in both cell fractions (Fig. 2A,B).

### INHBA, INHBB, CD49F, and FOLLISTATIN expression in main excretory ducts, and the weight and size of salivary grands after releasing main duct ligation

**Figure 3:**
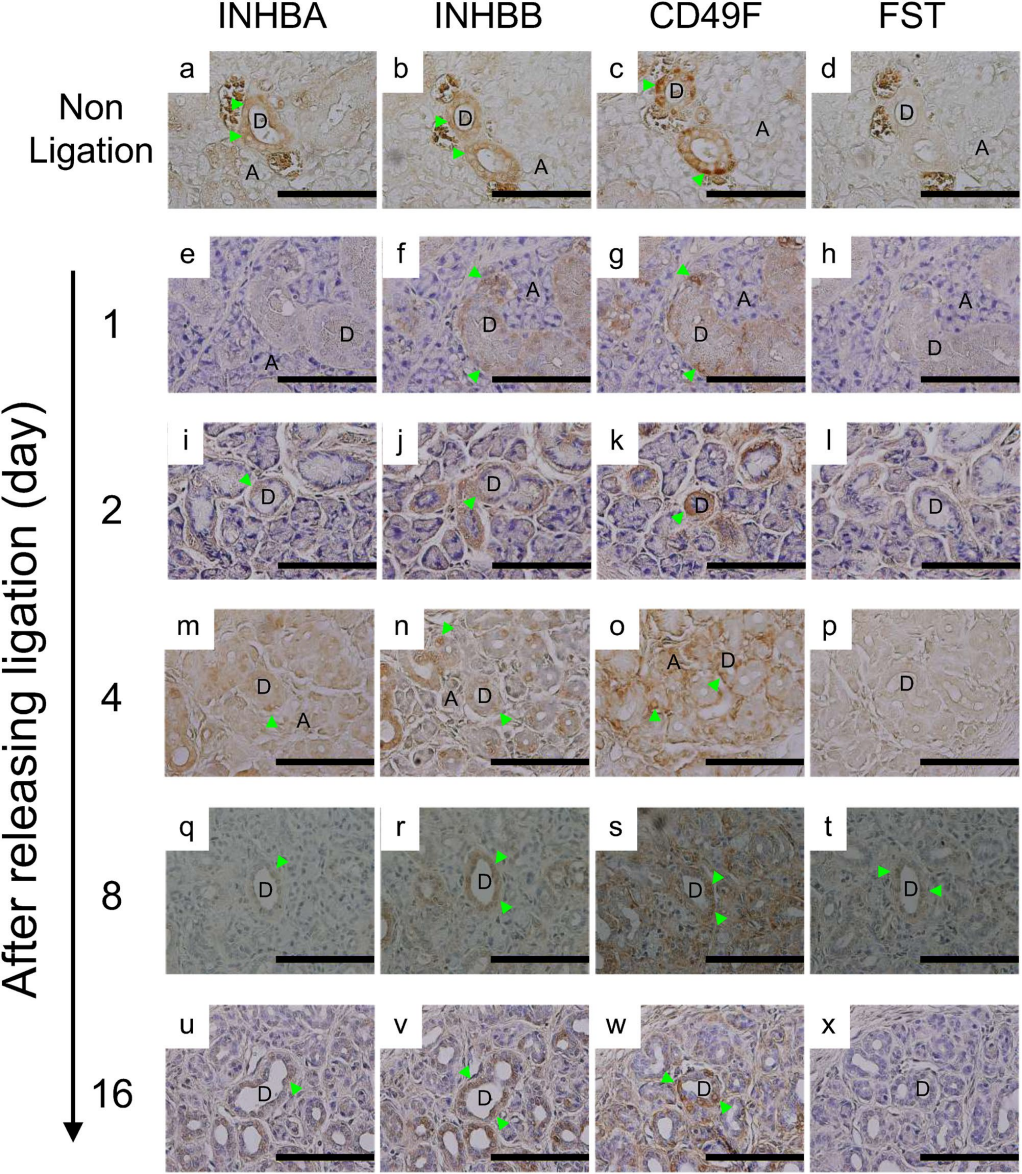
Immunohistochemical analysis of salivary glands for INHBA, INHBB, CD49F, and FST. Non-ligation side, (**a**)–(**d**); 1 day, (**e**)–(**h**); 2 days, (**i**)–(**l**); 4 days, (**m**)–(**p**); 8 days, (**q**)–(**t**), and 16 days after releasing ligation, (**u**)–(**x**). Typical images are shown from 3 independent experiments, and 1 experiment was performed using slides from paraffin blocks of salivary glands of 1 mouse. Arrow heads indicate cells expressing each protein. D: Duct. A: Acinar. Scale bar: 10 μm.

INHBA, INHBB, and CD49F were expressed in the duct epithelial cells of the non-ligation side, but follistatin was not detected. On the other hand, in the ligation side, INHBB and CD49F were expressed for all observation days after releasing the main duct ligation. In contrast, INHBA was not detected on any of the observation days. Interestingly, follistatin was not expressed on day 1, 2, and 4 after releasing the main duct ligation, but on day 8, follistatin was expressed in the duct epithelial cells, and decreased on day 16 (Fig. 3, Supplementary Figure 3). To investigate the correlation between follistatin expression pattern and the weight of salivary glands after the release of main duct ligation, we measured the weight, but no significant difference was observed (Supplementary Figure 4). Moreover, the size of salivary glands of ligation side up to 8 days was smaller than that of non-ligation side, and the number of acinar cells decreased (Supplementary Figure 2).

### Cell property of CD49f^+^ cells derived from salivary glands

**Figure 4:**
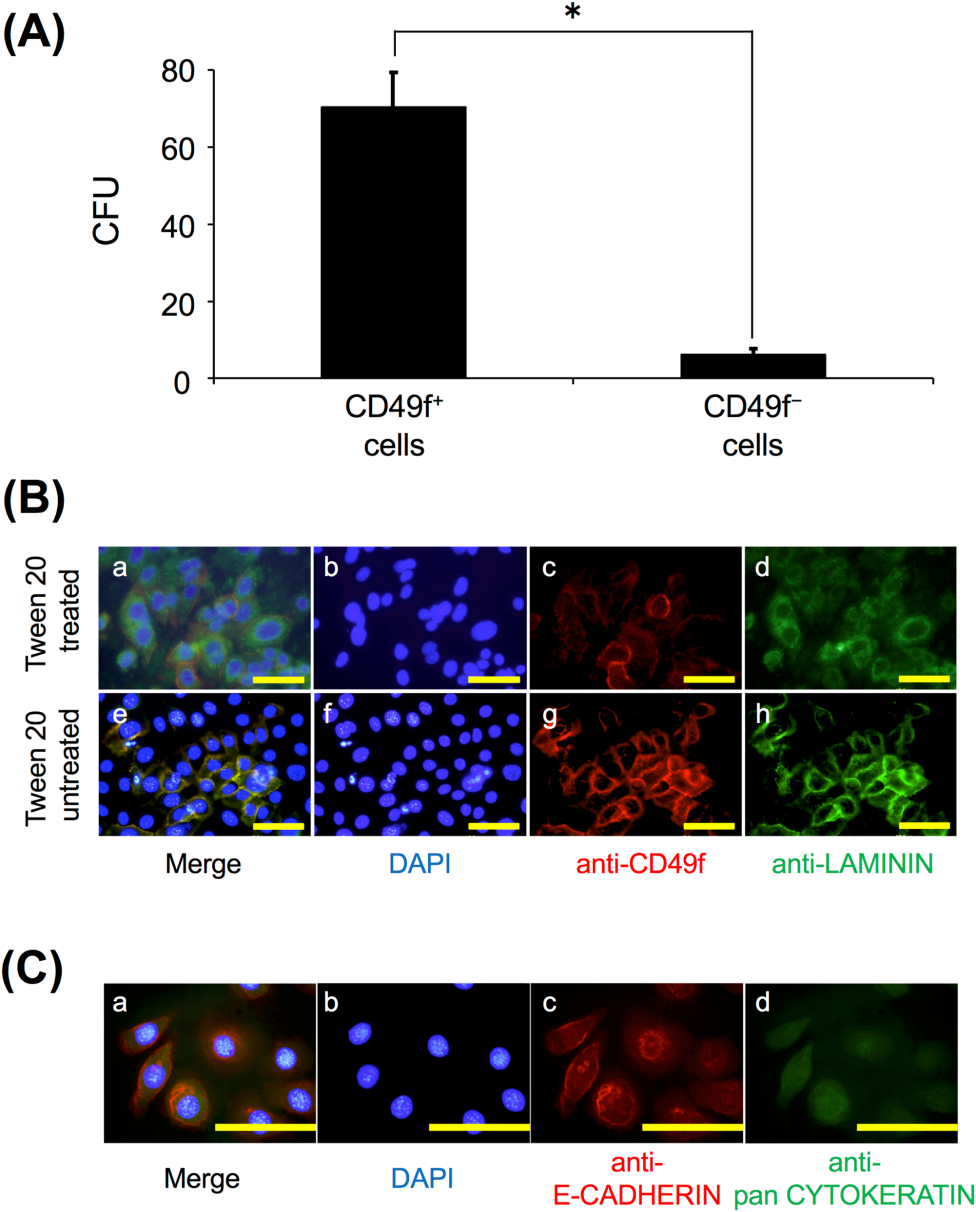
(**A**) Colony forming units (CFU) of cultured CD49f^+^ cells and CD49f^–^ cells at 8 days. The colonies consisting of more than 10 cells were counted. CD49f^+^ and CD49f^-^ cells fractionated from the salivary glands of 3 mice were used per experiment, and 3 independent experiments were carried out. *P < 0.05, Student’s *t* test. (**B**) Immunostaining of CD49f cell surface marker and laminin. Fluorescent immunocytochemistry was performed on sub-cultured CD49f^+^ cells with Tween-20 (upper row; for both cytoplasm and cell surface) or without (lower row; for cell surface) on the same sections. The CD49f marker was stained red in (c) and (g); laminin was stained green in (d) and (h); nuclei were stained blue with DAPI in (b) and (f). Overlaid images of 2 sets of 3 images are colored yellow in (a) and (e). The experiment was performed using sub-cultured CD49f^+^ cells fractionated from the salivary glands of 3 mice, and 3 independent experiments were carried out, and a typical set of images is shown. Scale bar: 10 μm. (**C**) Immunostaining of E-cadherin and pan-cytokeratin. Fluorescent immunocytochemistry was performed on the same sections of cultured CD49f^+^ cells. E-cadherin was stained red in (c); pan-cytokeratin was stained green in (d); nuclei were stained blue with DAPI in (b). Overlaid image of 3 images is colored yellow in (a). The experiment was performed using sub-cultured CD49f^+^ cells fractionated from the salivary glands of 3 mice; 3 independent experiments were carried out, and a typical set of images is shown. Scale bar: 10 μm.

The number of colony forming units (CFU) of cultured CD49f^+^ cells was remarkably higher than that of cultured CD49f^−^ cells (11.5-fold, *P* < 0.05) (Fig. 4A). Nucleus-cytoplasm ratio (N/C) of cultured CD49f^+^ cells was also high, and they presented a rounded spindle shape, while N/C of cultured CD49f^−^ cells was low, and they presented an expanded flat spread-out shape (Supplementary Figure 5A,B). Intracellular laminin, which was used as a pluripotency marker^19,20,24,26^, was expressed in cultured CD49f^+^ cells (Fig. 4B). Furthermore, albumin and α-1 fetoprotein, which were differentiation markers to the hepatic lineage, were also expressed in cultured CD49f^+^ cells and they could differentiate into cells of the hepatic lineage (Supplementary Figure 6). E-cadherin and pan cytokeratin (CK) were also expressed (Fig. 4C). These results indicated that cultured CD49f^+^ cells were the epithelial cells of salivary glands and had self-renewal and differentiation ability for the different cell lineage.

### Follistatin production in cultured CD49f^+^ cells

**Figure 5:**
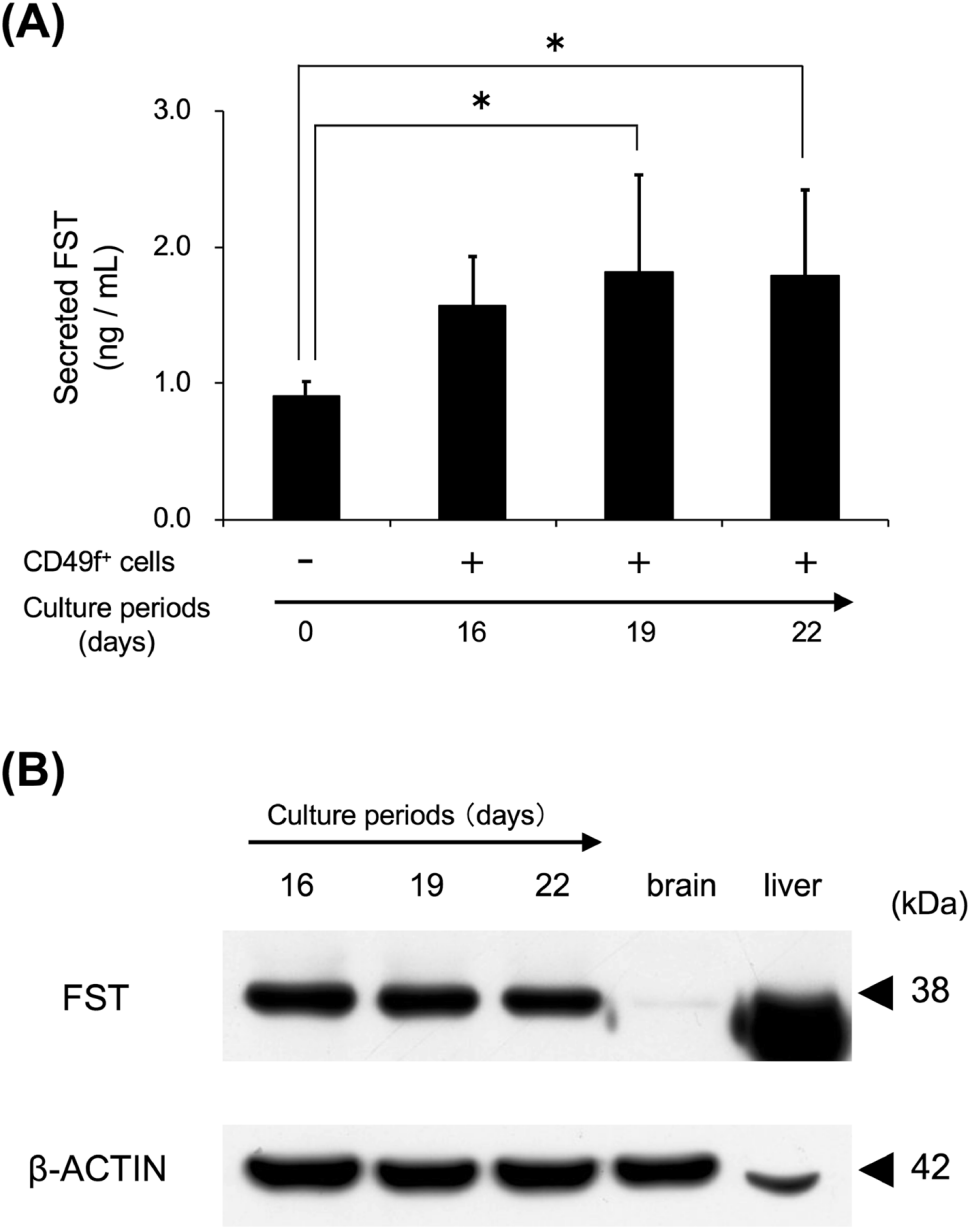
CD49f^+^ cells were cultured for 16, 19, and 22 days, and the supernatants and intracellular proteins were used in the following assays. (**A**) FST levels in the supernatants of cultured CD49f^+^ cells. FST amount at a specific culture period was measured by using ELISA. The experiment was performed using protein extracts of sub-cultured CD49f^+^ cells fractionated from the salivary glands of 3 mice, and 3 independent experiments were carried out. *P < 0.05, one-way ANOVA and Tukey’s HSD test. (**B**) Intracellular FST level in cultured CD49f^+^ cells. FST amount at specific culture period was detected by using western blotting (30 μg/lane). β-actin was used as an internal control by re-probing. Brain protein extracts and liver protein extracts were used as positive controls. Typical blotting images are shown in 3 independent experiments, and the experiment was performed using sub-cultured CD49f^+^ cells fractionated from the salivary glands of 3 mice.

Intracellular and secreted follistatin expression in cultured CD49f^+^ cells were examined using enzyme-linked immunosorbent assay (ELISA) and western blotting. The secreted follistatin level in the supernatant of cultured CD49f^+^ cells was significantly increased on days 19 and 22 (Fig. 5A). Intracellular follistatin was detected and a similar level maintained in cultured CD49f^+^ cells at days 16, 19, and 22, although it was not detected for freshly-isolated CD49f^+^ cells (Fig. 5B).

### Effects of follistatin gene knockdown by siRNA

**Figure 6:**
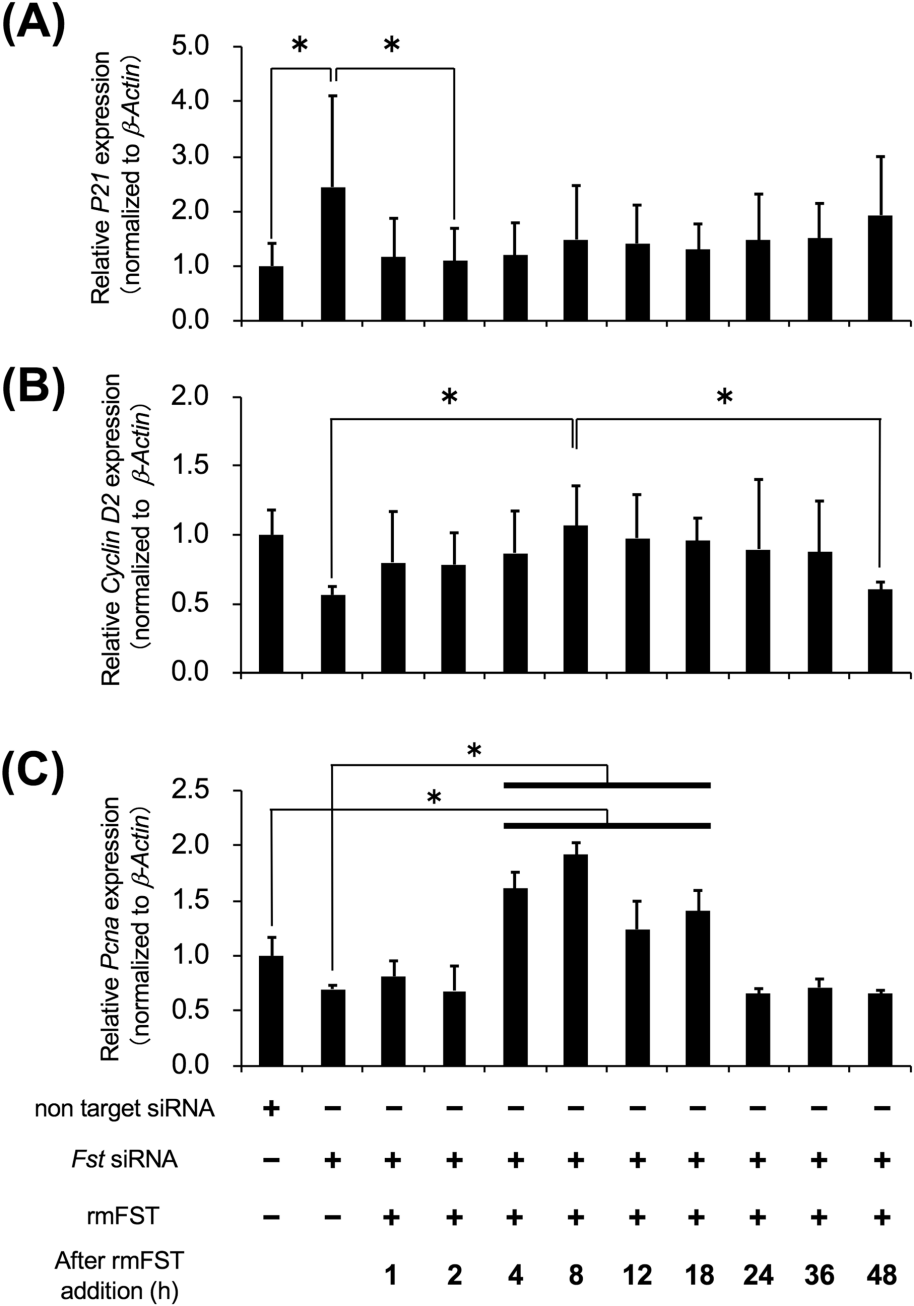
Effects of external FST in CD49f^+^ cells after knockdown of *Fst*. CD49f^+^ cells transfected with *Fst* siRNA were treated with recombinant mouse Follistatin (rmFST: 0.2 μg/mL). Non target siRNA was also used as a negative control. After specific periods of rmFST addition, the total RNA was recovered and used for RT-qPCR analysis. Levels of (**A**) *P21*, (**B**) *Cyclin D2*, and (**C**) *Pcna* mRNA expression normalized to *β-Actin* are shown. One experiment was performed using sub-cultured CD49f^+^ cells fractionated from the salivary glands of 3 mice, and 3 independent experiments were carried out. *P < 0.05, one-way ANOVA and Tukey’s HSD test.

Since activin is related to cell proliferation^27^, we examined mRNA expression related to 3 proliferation genes: *Cyclin-dependent kinase inhibitor 1A* (*P21*), *Cyclin D2*, and *Proliferating cell nuclear antigen* (*Pcna*). The CD49f^+^ cells were transfected by *Follistatin* siRNA (siRNA knockdown efficiency: 84.3 ± 11.4%; Supplementary Figure 7), and subsequently treated with recombinant mouse follistatin (rmFOLLISTATIN). Two hours after rmFOLLISTATIN treatment, *P21* mRNA expression was significantly lower than that of the no addition group (Fig. 6A). At 8 h after rmFOLLISTATIN treatment, the expression of *Cyclin D2* mRNA, the downstream of P21, was significantly high compared to that of the no addition group (Fig. 6B). Moreover, *Pcna* mRNA expression at 4, 8, 12 and 18 h after rmFOLLISTATIN treatment was significantly high compared to that of the no addition group (Fig. 6C).

## Discussion

Several studies indicate that CD49f could be stem cell marker^19,20,28^. Accordingly, in this study, we assumed that CD49f^+^ cells are somatic stem cells or progenitor cells, and then investigated the properties of CD49f^+^ cells in mouse salivary glands. It is reported that the ratio of hematopoietic stem cells in mouse bone marrow is approximately 0.004%^9^ and that of neural stem cells in the subventricular zone in young adult mouse is approximately 1.0%^29^. These findings suggest that somatic stem cells are rarely found in organs, however, the ratio of freshly isolated CD49f^+^ cells in salivary glands was 12.1 ± 1.1% (Fig. 1A) and the rate was much higher than in other somatic stem cells. This result suggests that many different types of cells, except for somatic stem cells, are also contained in the CD49f^+^ subpopulation. We consider that it is not enough to use only CD49f as a stem cell marker to separate pure somatic stem cells, and it is essential to combine it with other stem cell markers. One group reported that salivary gland tissue-derived stem cells expressed high levels of CD49f and c-Kit^30^. Therefore, one of the candidates for stem cell markers may be c-Kit. However, another group reported recently that c-Kit positive cells in adult salivary glands do not function as tissue stem cells^31^. Therefore, there is the possibility that CD49f^+^ and c-Kit negative cells are stem cells. Other groups reported that myoepithelial cells of some tissue also express CD49f^32–34^. CD49f^+^ cells in this study may also include myoepithelial cells. In that case, smooth muscle actin (SMA), which is myoepithelial cells marker, may be useful for the isolation of somatic stem cells in adult salivary glands. Moreover, we need to characterize CD49f^+^ cells population more precisely by RT-qPCR using other stem cell markers; Sca-1, CD133, and lineage markers; CK14 (basal/myoepithelial cell marker), CK18 (ductal cell marker), or SMA for the separation of stem cells population. Future study in our research direction should incorporate these matters.

To confirm the cell property of CD49f^+^ cells, we examined their self-renewal and multipotent abilities in vitro. Several studies reported that CFU assay is carried out for checking self-renewal ability^35–38^ and the expression of intracellular laminin is used for verifying multipotent ability^19,20,24,26^. This study revealed that the number of CFUs in CD49f^+^ cells was remarkably higher than that in CD49f^−^ cells (Fig. 4A), that CD49f^+^ cells expressed intracellular laminin (Fig. 4B), and that these cells differentiated into the cells which express albumin and α-1 fetoprotein, which are differentiation markers for the hepatic lineage (Supplementary Figure 6). It is also known that CD49f binds to laminin. Meanwhile, we detected the extracellular laminin expression of some CD49f^+^ cells. This result may suggest that several CD49f^+^ cells produce extracellular laminin by themselves to mediate cell–cell contact between CD49f^+^ cells and laminin. Moreover, sporadically, some cultured CD49f^+^ cells presented no expression of CD49F protein. This result implies that CD49f^+^ cells may differentiate into other cell types and lose multipotency during in vitro culture.

The previous report indicated that intercalated duct cells differentiate into acinar cells and granular duct cells from 1 to 2 weeks using 3H-thymidine incorporation into DNA^13^. Furthermore, another study implies that CD49f is only expressed in excretory ducts of mouse salivary glands^23^. Therefore, we confirmed that CD49F protein expression was only located in the duct epithelial cells of mouse salivary glands (Fig. 3), and that CD49f^+^ cells expressed E-cadherin and pan-cytokeratin (Fig. 4C). Considering that CD49f is a candidate as a stem cell marker, there is the possibility that CD49f^+^ cells are somatic stem cells in duct epithelium of mouse salivary glands and have the ability to participate in regeneration and remodeling of salivary glands.

In this study, we focused CD49f^+^ cells-derived growth factors to verify the properties of CD49f^+^ cells and selected *Inhbb* from the analyzed results of our PCR array (Fig. 1B). Inhibin βA and βB subunits, encoded by *Inhba* and *Inhbb*, respectively, are homo- or hetero-dimerized to yield activins. They stimulate the secretion of follicle stimulating hormone (FSH)^39^. Inhibins, which are pituitary FSH secretion inhibitors and inhibit the activin effects by binding to activin’s receptor competitively^40^, are composed of inhibin α and βA or βB subunits, encoded by *Inha*, *Inhba*, and *Inhbb*, respectively. Follistatin has a similar effect as inhibin, by inhibiting the signaling pathway of activin through the inhibition of the binding of activin to the receptors, by forming an activin-follistatin complex^41^.Therefore, in mRNA and protein levels, we examined the expression levels of these 4 growth factors; inha, inhba, inhbb, and follistatin. *Inhba*, *Inhbb*, and *Follistatin*, and INHBA and INHBB expression levels in freshly-isolated CD49f^+^ cells were higher than those in CD49f^−^ cells, but for *Inha* mRNA expression, there were no significant differences (Figs. 1C, 2A, B). Cultured CD49f^+^ cells produced follistatin (Fig. 5A,B) although follistatin was not detected in freshly-isolated CD49f^+^ cells (Fig. 2A,B).

Next, we examined mRNA expression of *P21*, *Cyclin D2*, and *Pcna* because it is also known that activin belongs to transforming growth factor beta (TGF-β) superfamily and arrests cell proliferation in the G0-G1 cell cycle phase^27^. Regarding mRNA levels, we showed that those of *P21* were increased and those of *Cyclin D2* mRNA were decreased by *Follistatin* siRNA; however, those of *P21* were decreased and those of *Cyclin D2* and *Pcna* were increased by the addition of recombinant mouse follistatin (Fig. 6A–C). In cultured CD49f^+^ cells, we also checked the mRNA expression of activin receptors, *Acvr2a* and *Acvr2b,* and cultured CD49f^+^ cells expressed both receptors (Supplementary Figure 9). This result implies that activins produced by CD49f^+^ cells bind to their receptors on CD49f^+^ cells, and activin pathway occurs^42^. These results suggest that freshly-isolated CD49f^+^ cells express activin but not follistatin in mRNA and protein level, and activin caused G1 arrest and growth inhibition. After sub-culturing CD49f^+^ cells, they not only expressed activin, but also follistatin at an mRNA and protein level, and activin pathway was blocked and G1 arrest was released by follistatin. In consequence, the cell cycle of CD49f^+^ cells transited into the S phase and then CD49f^+^ cells started proliferating. This study indicated an opposite mRNA expression of *Cyclin D2* and *P21*. The reason behind this is that cyclin D2 positively regulates cyclin dependent kinase 4 (CDK4) activity by forming a binary complex with CDK4; by contrast, P21 negatively regulates kinase activities of both CDK4 and CDK2 by binding with cyclin D-CDK4 and cyclin E-CDK2, respectively. From the above, we suggest that the control of the proliferation of CD49f^+^ cells is made through the interaction between activin and follistatin (activin-follistatin axis). The alteration of the molecular axis has a crucial role in the regulation of the regeneration of mouse salivary glands. However, it is unclear whether the interaction between activin and follistatin on cultured CD49f^+^ cells absolutely occurs in this study. We will examine whether CD49f^+^ cells express ACVR2A or ACVR2B in protein level by western blotting, and whether recombinant follistatin protein can affect phosphorylation of R-SMAD, that is activation of activin pathway, in the future research.

To confirm the expression of activin and follistatin in vivo, we used acinar apoptosis models of mouse salivary glands induced by main duct ligation. INHBA and INHBB were expressed for all days and follistatin was expressed only at the 8-day point after releasing the main duct ligation (Fig. 3). However, the glandular system of salivary glands was observed to be hardly recovered in histological images using hematoxylin–eosin staining (Supplementary Figure 2) and the weight of salivary glands did not return to the original weight (Supplementary Figure 4). These data indicate that salivary glands did not fully regenerate, suggesting that the activin-follistatin axis alone is insufficient or that follistatin expression is not enough to regenerate salivary glands. However, we don’t have the evidence that CD49f^+^ cells absolutely express these proteins in vivo. In the next step, we will perform multiple immunofluorescence staining. Moreover, lobules sometime have duct-like structure after ligation. Accordingly, we will carry out multiple immunofluorescence staining to clarify the distribution of CD49f^+^ cells using CK14, CK18, and SMA as our next stage study.

The liver is known to have high regenerative ability, and in vitro rat hepatocytes produce activin and follistatin for proliferation^43^. Furthermore, activin is continuously produced in the liver and follistatin administration promotes liver regeneration in rat hepatectomy models^44,45^. On the other hand, the kidney is known to have a low regenerative ability, and activin expression in the damaged kidney increases notably and follistatin administration improves kidney function, based on histological findings in kidney^46^. These results suggest that the activin-follistatin axis may be associated with liver and kidney regeneration and restoration. Moreover, the activin-follistatin axis has important roles in other organs and early embryogenesis^47–49^. Therefore, further in vivo assays would be essential to investigate the role of the activin-follistatin axis in salivary glands.

**Figure 7:**
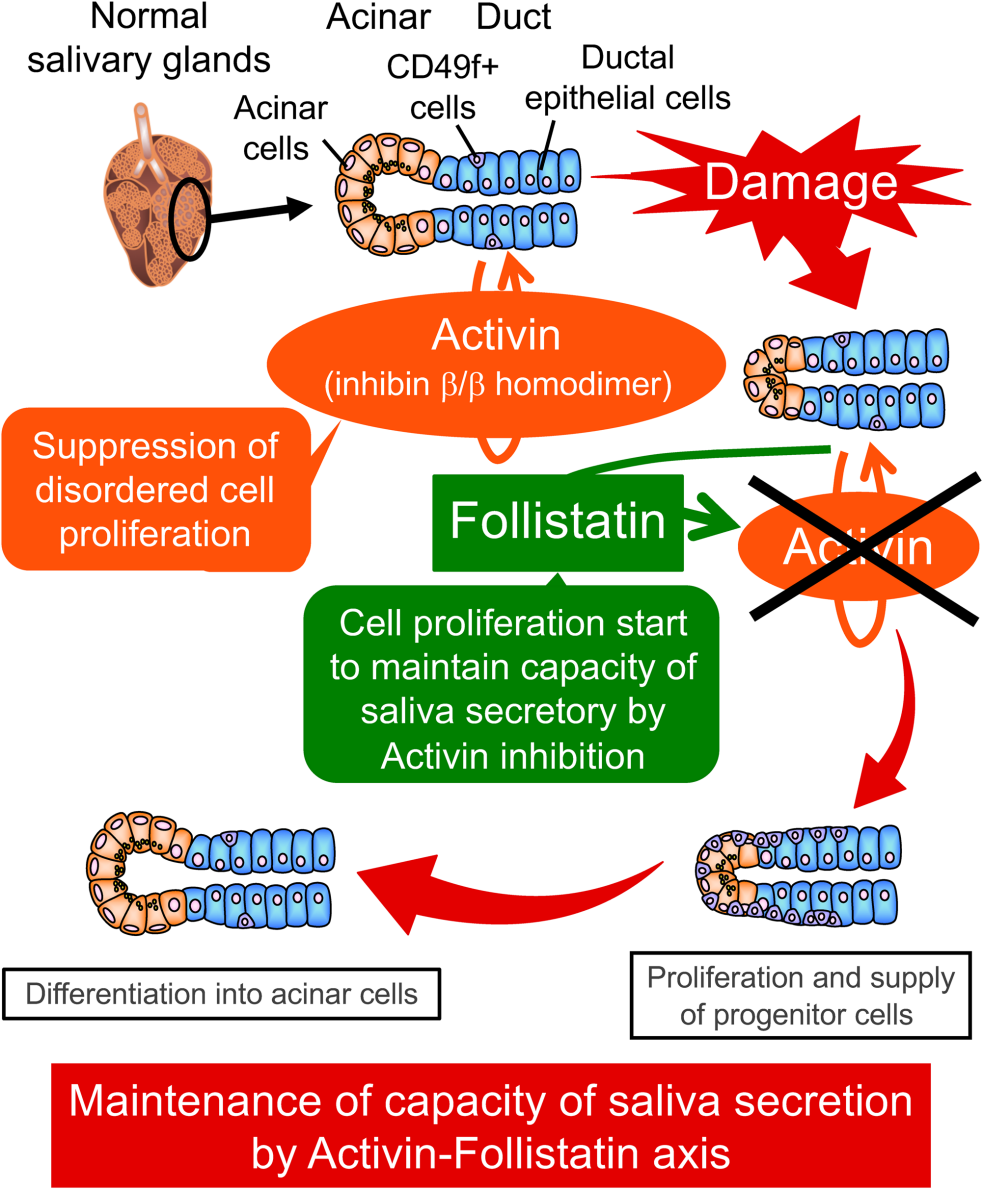
Hypothetical model for the activin-follistatin axis in the CD49f^+^ cells: to suppress the disordered cell proliferation, DNA synthesis is usually suppressed through G1 phase arrest by activin, which is produced and secreted by CD49f^+^ cells located in the salivary glands. However, once salivary glands are damaged, CD49f^+^ cells produce and secrete follistatin, and then start cell proliferation through the abrogation of G1 phase arrest by a decrease of P21 expression to maintain the secretory capacity of saliva.

In summary, our results highlighted that fleshly-isolated CD49f^+^ cells expressed inhba and inhbb, which are both activin components, and cultured CD49f^+^ cells began to express follistatin, which has the ability of activin inhibition in mRNA and protein levels. In vivo, follistatin was expressed in mechanically damaged salivary glands. These results suggest that CD49f^+^ cells express activin in non-damaged salivary glands, which may suppress a disordered proliferation of CD49f^+^ cells. However, when salivary glands are mechanically damaged, CD49f^+^ cells produced follistatin and may proliferate and supply progenitor cells through activin-follistatin axis (Fig. 7). In other words, there is the possibility that the proliferation of CD49f^+^ cells through activin-follistatin axis are related to repair/regeneration in male mouse salivary glands. We will try to confirm this character of CD49f^+^ cells as a part of stemness in our future study.

It is well known that submandibular glands of male rodents have GCT which female rodents don’t have, and GCT secret many kinds of growth factors and bioactive substances^25^. This character of GCT could be beneficial for the isolation of growth factors and their related factors required for the repair of salivary gland. Thus, we focused on only male mice which have GCT. Indeed, male mice salivary glands are not similar to human salivary glands because of GCT. However, we believe that we may have opened the gate for the repair of mature salivary gland. Further studies using female mouse is needed to confirm our result. Moreover, A further understanding of the activin-follistatin axis will help to elucidate proliferation in regeneration and restoration of not only salivary glands, but also many other organs, and even embryogenesis.

## Material and methods

### Animals

Five-week-old male C57BL/6 mice were purchased from CLEA Japan (Tokyo, Japan). Animal care was performed in accordance with the Guidelines for the Treatment of Experimental Animals, and all experiments were approved by the Animal Care and Use Committee of Okayama University (# OKU-2014168).

### Isolation and culture of cells derived from salivary glands

For the isolation of cells from the cell suspension, all centrifugations were performed at 120 × *g* for 5 min at room temperature (RT). Five-week-old male mice were sacrificed and both submandibular glands were excised, minced, and incubated in 1 mL of ethylene glycol-bis (β-aminoethylether)-N,N,N’,N’-tetraacetic acid (EGTA) buffer at 37 °C for 20 min, and then centrifuged. The cell pellets were resuspended in 1 mL of Dulbecco’s modified Eagle medium/F12 1:1 (D-MEM/F12 1:1, Life Technologies, Carlsbad, CA, USA) containing 1.67 mg/mL collagenase (Worthington Biochemical, Lake Wood, NJ, USA) and 1.33 mg/mL hyaluronidase (Sigma-Aldrich, St. Louis, MO, USA), and incubated for 40 min at 37 °C. After centrifugation, the remaining tissue was further treated with D-MEM/F12 1:1 containing 1.67 mg/mL Dispase II (Sanko Junyaku, Tokyo, Japan) for 60 min at 37 °C. The cell suspension was passed through a 70-μm Cell Strainer (BD Biosciences, Franklin Lakes, NJ, USA), and centrifuged. The pellets were resuspended in 10 mL of keratinocyte serum-free medium (K-SFM; Life Technologies). Subsequently, the cells were fractionated using magnetic cell separation (MACS; Miltenyi Biotec, Auburn, CA, USA), as described below, and these freshly-isolated CD49f^+^ and CD49f^−^ cells were immediately used for some experiments. For other experiments, CD49f^+^ and CD49f^−^ cells were plated on a 60-mm culture dish (Corning, Corning, NY, USA) at 1.0 × 10^4^ cells/cm^2^ with K-SFM containing 100 U/mL penicillin G and 100 μg/mL streptomycin (Life Technologies). The cells were cultured at 37 °C, 5% CO_2_, and the medium was renewed 7 days after cell seeding, and then renewed every 3 days. The shape of cultured CD49f^+^ and CD49f^−^ cells was observed under a microscope (ECLIPSE TS100, Nikon, Tokyo, Japan). For induction of the hepatic lineage, CD49f^+^ cells were plated (1.0 × 10^4^ cells/well) on a 1:3-thick Matrigel (BD Biosciences) in 12-well dishes (1.5 mL/well) in K-SFM medium with antibiotics, which was additionally supplemented with 10 mmol/L nicotinamide.

### Magnetic cell separation (MACS)

The cells were incubated with an anti-CD49f antibody (0.1 μg/1.0 × 10^6^ cells; clone: GoH3, AbD Serotec, Oxford, UK) for 5 min on ice, followed by reaction with goat anti-rat IgG MicroBeads (Miltenyi Biotec) for 5 min on ice. The cells were subsequently passed through a 35-μm Cell Strainer (BD Biosciences), and separated into CD49f^+^ and CD49f^−^ cell fractions using an autoMACS (Miltenyi Biotec) and the separation program (POSSEL separation program, Miltenyi Biotec).

### Flow cytometry

The cells isolated from salivary glands were reacted with the fluorescein-5-isothiocyanate (FITC)-conjugated CD49f antibody (clone: GoH3, Miltenyi Biotec Inc.) for 10 min at 4 °C, and FITC-conjugated isotype control antibody (eBioscience, San Diego, CA, USA) was used as a negative control. A total of 1.0 × 10^4^ cells was used for each analysis by using FACScan (BD Biosciences) and CellQuest (BD Biosciences).

### Profiling of growth factors mRNA expression

Total RNAs of CD49f^+^ and CD49f^−^ cells from freshly-isolated and sub-cultured CD49f^+^ cells were respectively isolated using a RNeasy Mini Kit (QIAGEN, Hilden, Germany). RNA concentration and quality (A260/A280 ≈ 2.0; A260/A230 ≈ 2.0) were determined using a NanoDrop 2000 (Thermo Fisher Scientific, Waltham, MA, USA). One microgram of total RNA extracted from freshly-isolated CD49f^+^ and CD49f^−^ cells was amplified using Applied Biosystems 7300 Real-Time PCR System (Life Technologies), and the RNA expression levels between these cells were compared using a Mouse Growth Factors RT^2^ Profiler PCR Array (QIAGEN). Obtained data were analyzed using the free Web-based PCR Array Data Analysis Software (https://dataanalysis.sabiosciences.com/pcr/arrayanalysis.php, QIAGEN).

### mRNA expression analysis by using reverse transcribed-real time PCR (RT-qPCR)

cDNA was synthesized with 1 μg of the total RNA using SuperScript VILO MasterMix (Life Technologies). cDNA was quantified by using qPCR with specific primers (Supplementary Table 1), and the levels were calculated using the ΔΔCt method. *Glucuronidase beta* (*Gusb*) and *β-Actin* were used as internal controls.

### Western blotting

Total cell lysates were respectively extracted from CD49f^+^ and CD49f^−^ freshly-isolated cells, and CD49f^+^ cells cultured for 16, 19, and 22 days with a radio-immunoprecipitation buffer containing a protease inhibitor cocktail (Roche Applied Science, Indianapolis, IN, USA). The total protein was measured using an RC DC Protein Assay Kit (Bio-Rad Laboratories, Richmond, CA, USA). After the separation of 30 µg of protein using a denaturing 12% polyacrylamide gel and transferal to a polyvinylidene difluoride membrane (Millipore, Billerica, MA, USA), the membrane was blocked with 5% skim milk in TTBS (10 mM Tris–HCl, pH 7.4, 150 mM NaCl, 0.05% Tween-20), and incubated with an anti-Inhba monoclonal antibody (1:5,000; abcam, Cambridge, UK), an anti-Inhbb monoclonal antibody (1:5,000; abcam), an anti-Follistatin polyclonal antibody (1:1,000; abcam), and an anti-β-Actin monoclonal antibody (1:10,000; Sigma-Aldrich), followed by horseradish peroxidase (HRP)-conjugated secondary antibodies (GE Healthcare, Buckinghamshire, UK). Membranes were reused after re-probing using Restore Western Blot Stripping Buffer (Thermo Fisher Scientific). The blots were scanned and analyzed using Image J (Version 1.46r, National Institutes of Health, Bethesda, MD, USA). β-ACTIN intensity was used as an internal control.

### Duct ligation mouse model

After submandibular glands at both sides of five-week-old male mice were exposed, one main secretory duct was ligated with the clip (Micro Clamp B-1 3.5 × 1.0; Fine Science Tools, North Vancouver, B.C. Canada), and another side was not ligated and was used as a control. Six days later, the submandibular glands were re-exposed, and the clips were removed. At 1, 2, 4, 8, 16 days after their removal, each salivary gland was excised, weighed, and photographed.

### Immunohistochemical staining

Submandibular glands of duct ligation mouse model were fixed in phosphate-buffered saline (PBS) containing 4% paraformaldehyde (4% PFA-PBS), and embedded in paraffin. Sections were sliced in 4-μm thick slices for immunohistochemical staining and hematoxylin–eosin staining. The sections were incubated with anti-Inhibin beta A antibody (1:100; Bioss, Boston, MA, USA), anti-Inhibin beta B antibody (1:100; Bioss), anti-CD49f antibody (1:250; abcam), and anti-Follistatin antibody (1:100; Bioss), followed by the reactions with SuperPicture Polymer Detection Kit (abcam) and hematoxylin. The stained sections were observed under a microscope (DP70; OLYMPUS, Tokyo, Japan).

### Colony forming unit analysis

CD49f^+^ and CD49f^−^ cells were plated into 6-well plates (Corning) at a density of 2.5 × 10^4^ cells/cm^2^. The cells were cultured for 8 days in K-SFM, as mentioned above. Colonies that consisted of more than 10 cells were counted under a microscope (ECLIPSE TS100). Their colony forming ability was the mean value of the total number of colonies counted in 3 wells, and the assay was performed 3 times independently.

### Immunocytochemistry

CD49f^+^ and CD49f^−^ cells were cultured in Lab-Tek II Chamber Slide (Thermo Fisher Scientific) at a 1.0 × 10^4^ cells/cm^2^ density for 7 days with K-SFM, as mentioned above. Subsequently, the cultured cells were fixed with 4% PFA-PBS for 15 min at 37 °C, followed by blocking with 10% Non-Immune Goat Serum (Life Technologies) or Donkey Serum (Sigma-Aldrich) for 30 min at RT. Slides were then treated in PBS containing 0.2% Tween-20 for 5 min at RT. On the other hand, control slides were washed only using PBS. Regarding CD49f^+^ cells after induction of hepatic lineage, the Matrigel Matrix was depolymerized using a Cell Recovery Solution (Corning), and spheres were solidified in iPGell (Genostaff, Tokyo, Japan), fixed in 4% PFA-PBS, embedded in paraffin, and sliced in 4-µm-thick slices. Subsequently, the cells were incubated with an anti-CD49f (10 μg/mL; clone: GoH3, AbD Serotec), an anti-Laminin (1:50; DAKO, Glostrup, Denmark), an anti-E-cadherin (1:200; Cell Signaling Technology, Danvers, MA, USA), an anti-pan Cytokeratin (1:200; abcam), an anti-Albumin (1:200; Novus Biologicals, Littleton CO, USA), and an anti-alpha 1 Fetoprotein antibodies (1:100; AFP) (Bioss), for 30 min at RT, followed by incubation with Alexa 594-labeled anti-rat IgG (1:500), Alexa 488-labeled anti-rabbit IgG (1:500), Alexa 594-labeled anti-rabbit IgG (1:500), Alexa 594-labeled anti-mouse IgG (1:500), and Alexa 594-labeled anti-goat IgG (1:500; all were from Life Technologies) for 30 min at RT after washing 3 times with PBS. Subsequently, they were mounted using VECTASHIELD Mounting Medium with 4′,6-diamidino-2-phenylindole (DAPI) (Vector Laboratories, Inc., Burlingame, CA, USA). Cells were observed under a fluorescence microscope (DP70; OLYMPUS).

### Enzyme-linked immunosorbent assay (ELISA)

CD49f^+^ cells were cultured for 16, 19, and 22 days. Follistatin in culture supernatants was measured using an ELISA kit (Uscn Life Science, Wuhan, China).

### Small interfering-RNA (siRNA) and addition of follistatin protein

CD49f^+^ cells were plated on 6-well plates at 3 × 10^5^ cells/well. After 48 h, CD49f^+^ cells were transfected with *Follistatin* siRNA (GE Healthcare Dharmacon, Lafayette, CO, USA) or Non-Targeting siRNA Pool (GE Healthcare Dharmacon), and Lipofectamine RNAiMAX Reagent (Life Technologies), when cells reached a 50–60% confluence. Subsequently, 0.2 μg/mL Recombinant Mouse Follistatin protein (rmFOLLISTATIN: R&D Systems, Minneapolis, MN, USA) was added to each well. The total RNA was isolated from cells cultured for 1, 2, 4, 8, 12, 18, 24, 36, and 48 h after rmFOLLISTATIN addition. Effects of the addition of siRNA and rmFOLLISTATIN were evaluated by using RT-qPCR. Total RNA from CD49f^+^ cells transfected with a non-target control siRNA (Thermo Fisher Scientific) was used as a negative control.

### Statistical analysis

All results are represented as means ± SD from at least 3 independent experiments, as shown in each figure legend. Statistical analysis was determined by Student’s *t*-test or one-way analysis of variance (ANOVA) and Tukey’s honestly significant difference (HSD) test using the JMP 9 software (SAS institute, Cary, NC, USA). A *P*-value ≤ 0.05 was determined as statistically significant.

## Supplementary information

Supplementary Information.

## Data Availability

The datasets generated during in this study are available from the corresponding author on reasonable request.
