# Supplementary material for: Follistatin expressed in mechanically-damaged salivary glands of male mice induces proliferation of CD49f+ cells
Source: Sci Rep. 2020 Nov 17;10:19959. doi: 10.1038/s41598-020-77004-2 (PMC7673039; doi:10.1038/s41598-020-77004-2)
Supplement: Supplementary file 1 — Supplementary Information. [file 41598_2020_77004_MOESM1_ESM.pdf]

Follistatin Expressed in Mechanically-damaged Salivary Glands of Male Mice Induces Proliferation of CD49f^{+} Cells

A. Ikeda, T. Yamamoto, J. Mineshiba, S. Takashiba

Supplementary Figures and table Legends

Supplementary Table 1

PCR primers for RT-qPCR

Supplementary Figure 1.

RT-qPCR analysis for down-regulated genes is shown in Figure 1(B) in freshly-isolated CD49f cells: mRNA quantities of *Spp1* and *Gdf10* were determined relative to *Glucuronidase beta* (*Gusb*) by using the ΔΔCt method, and fold induction was shown. CD49f^{+} cells (solid bars), CD49f^{-} cells (open bars). One set of experiments was performed using the total RNA extracted from CD49f^{+} and CD49f^{-} cells fractionated from the salivary glands of 3 mice, and 3 sets of experiments carried out independently. *$P < 0.05$, Student's *t* test.

Supplementary Figure 2.

Macroscopic analysis of salivary glands (upper panels) and microscopic analysis of hematoxylin-eosin staining of the sections (lower panels) chronologically after releasing the ligation of main excretory ducts. The control sample had no ligation. Typical images are shown in 3 independent experiments, and 1 experiment was performed using a pair of salivary glands and one paraffin block from 1 mouse. Orange scale bar: 10 mm, and green scale bar: 1 mm.

Supplementary Figure 3.

Whole images of western blotting of intracellular FST protein in cultured $CD49f^+$ cells: 1 experiment was performed using protein extracts of sub-cultured $CD49f^+$ cells fractionated from the salivary glands of 3 mice, and 3 independent experiments (#1-3) were carried out, and blot #2 was used for Figure 5. See Figure 5 for details. M: molecular weight marker.

Supplementary Figure 4.

Weight of salivary glands after release of main duct ligation (control: no ligation side at opposite side in the same mouse). The experiment was performed using a pair of salivary glands from 1 mouse, and 3 independent experiments were carried out. N.S.: No significant difference, $^*P < 0.05$, one-way ANOVA and Tukey's HSD test.

Supplementary Figure 5.

(A) Cell colonies of $CD49f^+$ after 7, 10, 13, and 16 days.

(B) Cell shape of $CD49f^+$ and $CD49f^-$, cultured for 14 days.

Typical microscopic images are shown. One set of experiments was performed using $CD49f^+$ and $CD49f^-$ cells fractionated from the salivary glands of 3 mice were used per experiment, and 3 sets of experiments were carried out independently. Scale bar: 100 μm.

Supplementary Figure 6.

Immunostaining of albumin and α-1 fetoprotein in cultured $CD49f^+$ cells differentiated into the hepatic lineage: fluorescent immunocytochemistry was performed on $CD49f^+$ cells on the same slides. Albumin, red; α-1 fetoprotein,

green; DAPI, blue. Overlaid image of 3 images was colorized in yellow in (a). The experiment was performed using sub-cultured CD49f$^+$ cells fractionated from the salivary glands of 3 mice, and 3 independent experiments were carried out, and a typical set of images is shown. Scale bar: 10 μm.

Supplementary Figure 7.

Knock-down efficiency of *Fst* expression by siRNA. Total RNA was recovered at day 2 post-transfection and used for synthesis of cDNA (1 μg of total RNA). *Fst* mRNA expression was analyzed by using qPCR and shown after normalization to *β-Actin*. Non target siRNA was used as a negative control. The experiment was performed using sub-cultured CD49f$^+$ cells fractionated from the salivary glands of 3 mice, and 3 independent experiments were carried out. *$P < 0.05$, Student's *t* test.

Supplementary Figure 8.

Whole images of western blotting used in Figure 2(A). A representative set of blots of 3 independent experiments is shown. See Figure 2 for details.

Supplementary Figure 9.

Ct values of RT-qPCR analysis for *β-Actin*, *Acvr2a*, and *Acvr2b* in cultured CD49f$^+$ cells. One experiment was performed using sub-cultured CD49f$^+$ cells fractionated from the salivary glands of 3 mice, and 3 independent experiments were carried out.

Target genes	Primers	Product length (bp)
Csf1	F: 5’ - GGCATCATCCTAGTCTTGCTG - 3’<br>R: 5’ - TTCCACCTGTCTGTCCTCATC - 3’	147
Inhbb	F: 5’ - CGAGATCATCAGCTTTGCAG - 3’<br>R: 5’ - TGCACCACGAATAGGTTCTG - 3’	102
Tgfa	F: 5’ - TGGGTATCCTGTTAGCTGTGTG - 3’<br>R: 5’ - CTTGTTGAAGTGAGACACCACTG - 3’	98
Inhba	F: 5’ - ATCATCACCTTTGCCGAGTC - 3’<br>R: 5’ - GTTAGCCTTGGGGACTTTCAG - 3’	129
Inha	F: 5’ - GGCTATCCTTTTCCCAGCTAC - 3’<br>R: 5’ - GATGGCCGGAATACATAAGTG - 3’	108
Fst	F: 5’ - TCTTCTGGCGTGCTTCTTG - 3’<br>R: 5’ - CCTCTTCCTCCGTTTCTTCC - 3’	73
Gusb	F: 5’ - TCCAAGGGGTCAATAAGCAC - 3’<br>R: 5’ - CAACGGAGCAGGTTGAAATC - 3’	91
P-21	F: 5’ - GGAGCATGAATGGAGACAGAG - 3’<br>R: 5’ - ATCACCAGGATTGGACATGG - 3’	98
Cyclin D2	F: 5’ - CAGTGTGCATGTTCCTAGCTTC - 3’<br>R: 5’ - GTTCCACTTCAGCTTACCCAAC - 3’	143
Pcna	F: 5’ - TCACAAAAGCCACTCCACTG - 3’<br>R: 5’ - GCTTCCTCATCTTCAATCTTGG - 3’	136
Spp1	F: 5’ - GAGGAAACCAGCCAAGGTAAG - 3’<br>R: 5’ - ATCACTGCCAATCTCATGGTC - 3’	99
Gdf10	F: 5’ - AAGTACAACCGAAGAGGTGCTC - 3’<br>R: 5’ - GCTGTGAGGATCATTTCTGAGTC - 3’	140
Acvr2a	F: 5’ - ACCCTCCTGTACTTGTTCCTAC - 3’<br>R: 5’ - ATTGAGCAACTGGGCTTTCC - 3’	137
Acvr2b	F: 5’ - GTTCATTGCTGCCGAGAAAC - 3’<br>R: 5’ - GATGTTCCCCTTGAGGTAATCC - 3’	106
β-Actin	F: 5’ - CTTTTCCAGCCTTCCTTCTTG - 3’<br>R: 5’ - GGCATAGAGGTCTTTACGGATG - 3’	91

Supplementary Table 1

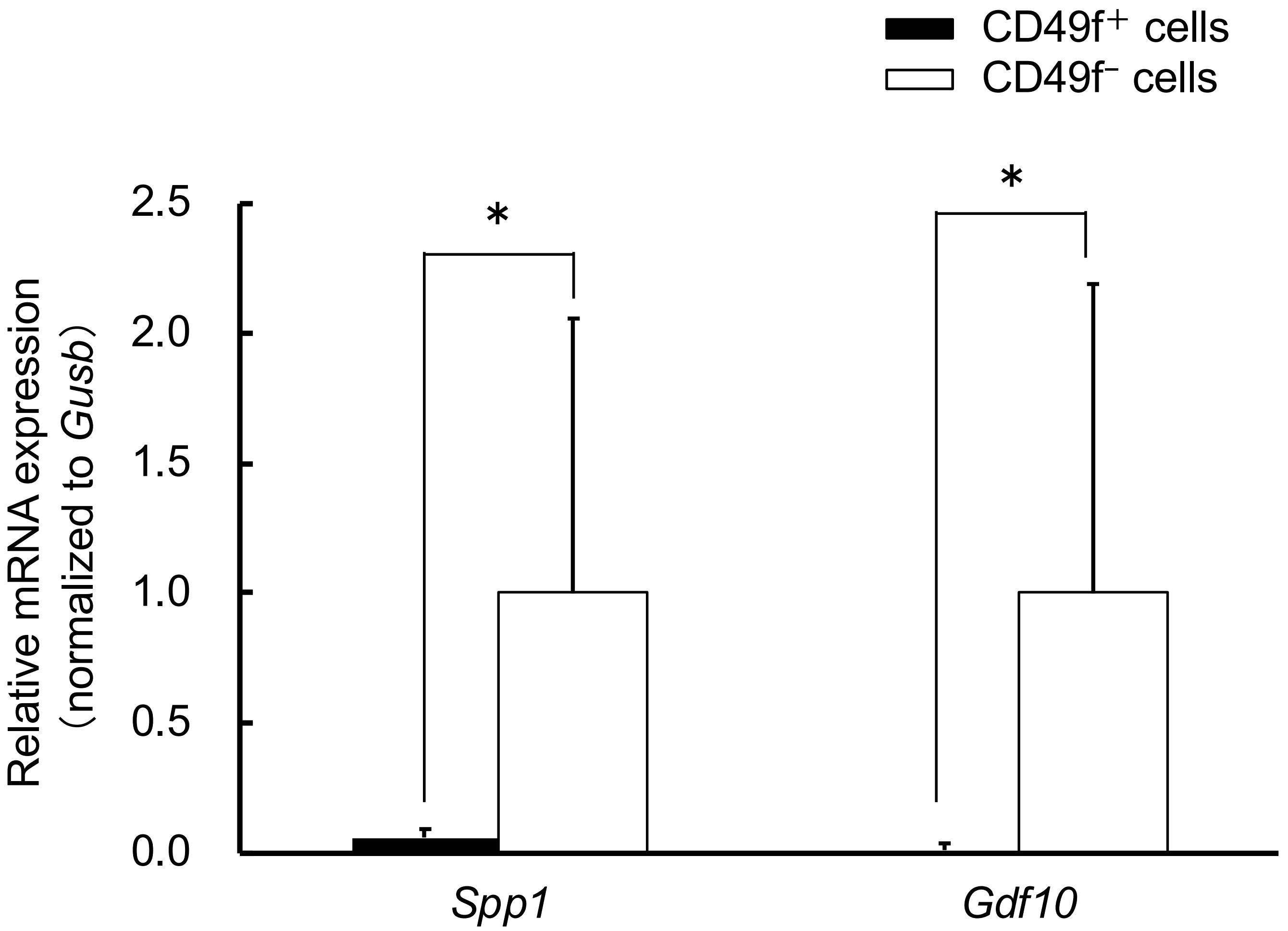

Supplementary Fig. 1

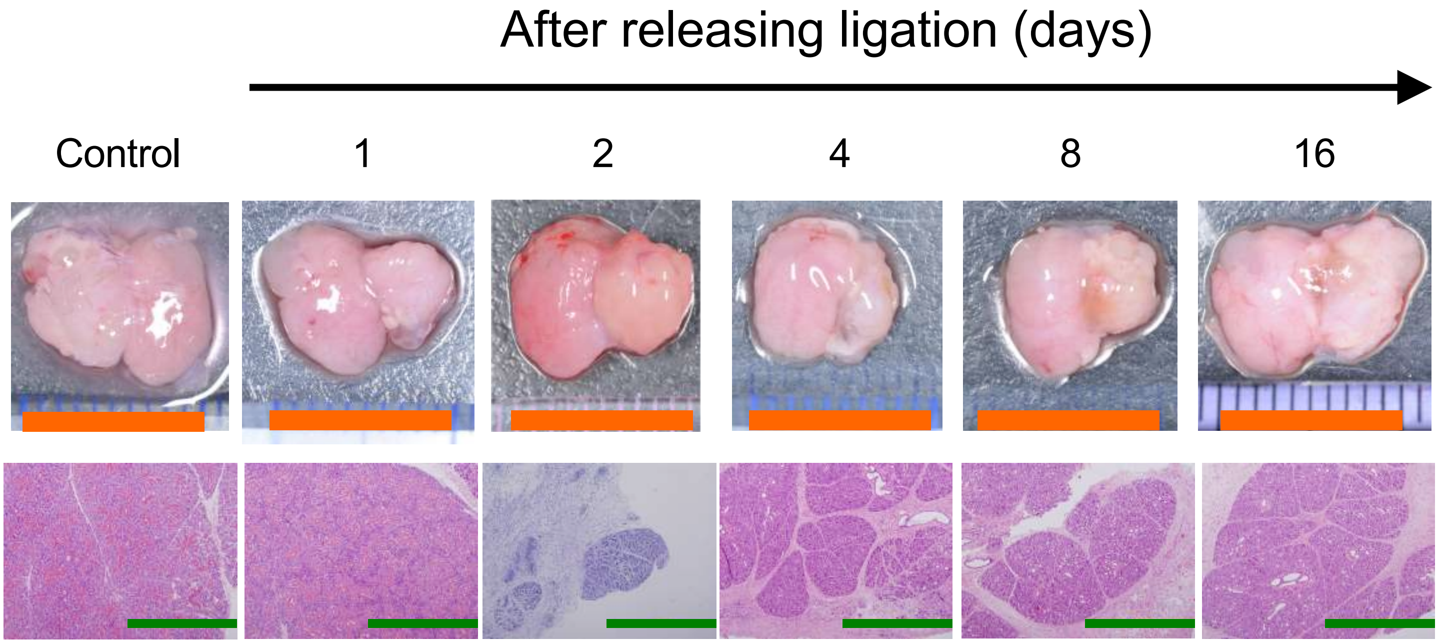

Supplementary Fig. 2

1

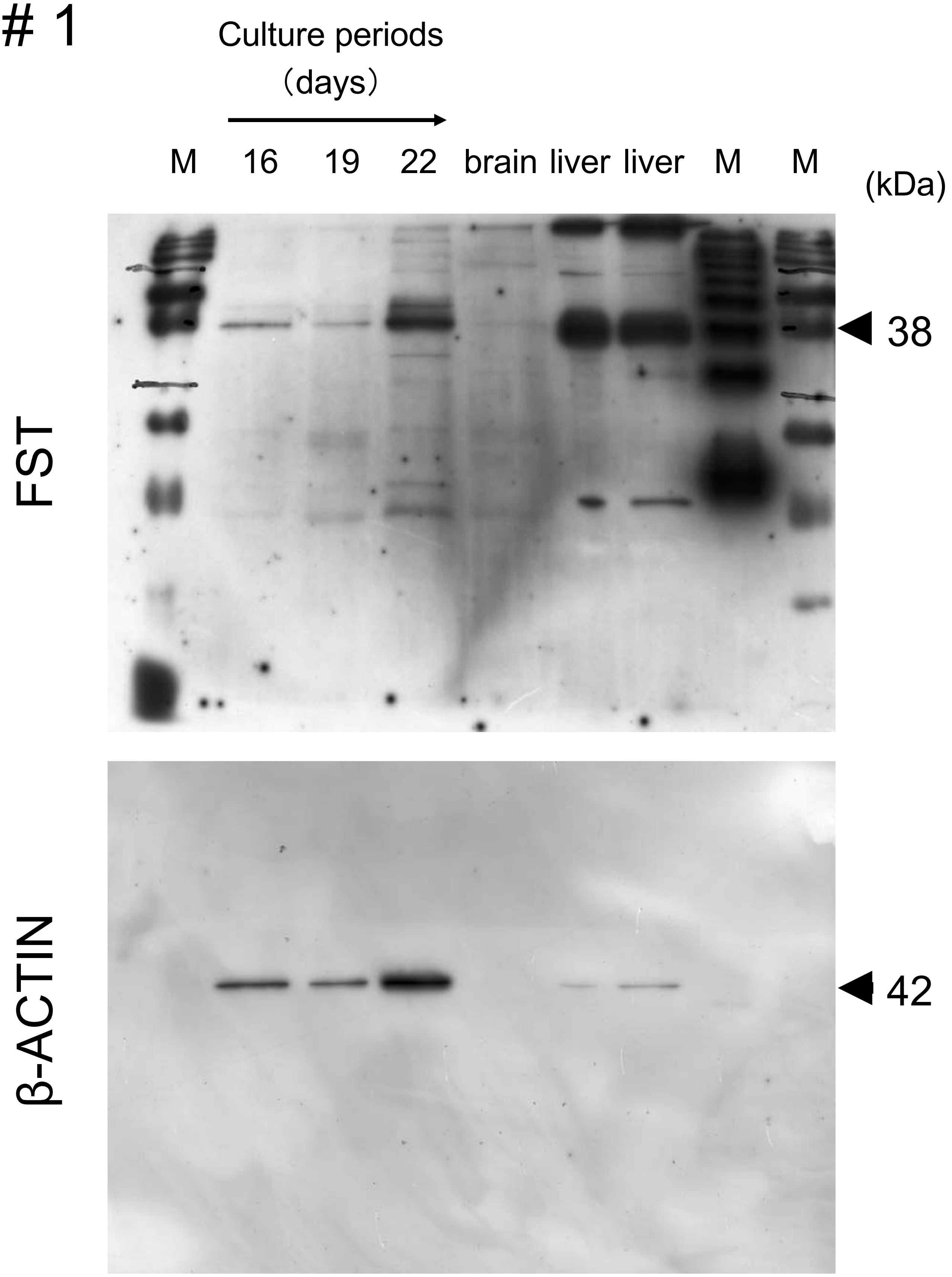

Supplementary Fig. 3

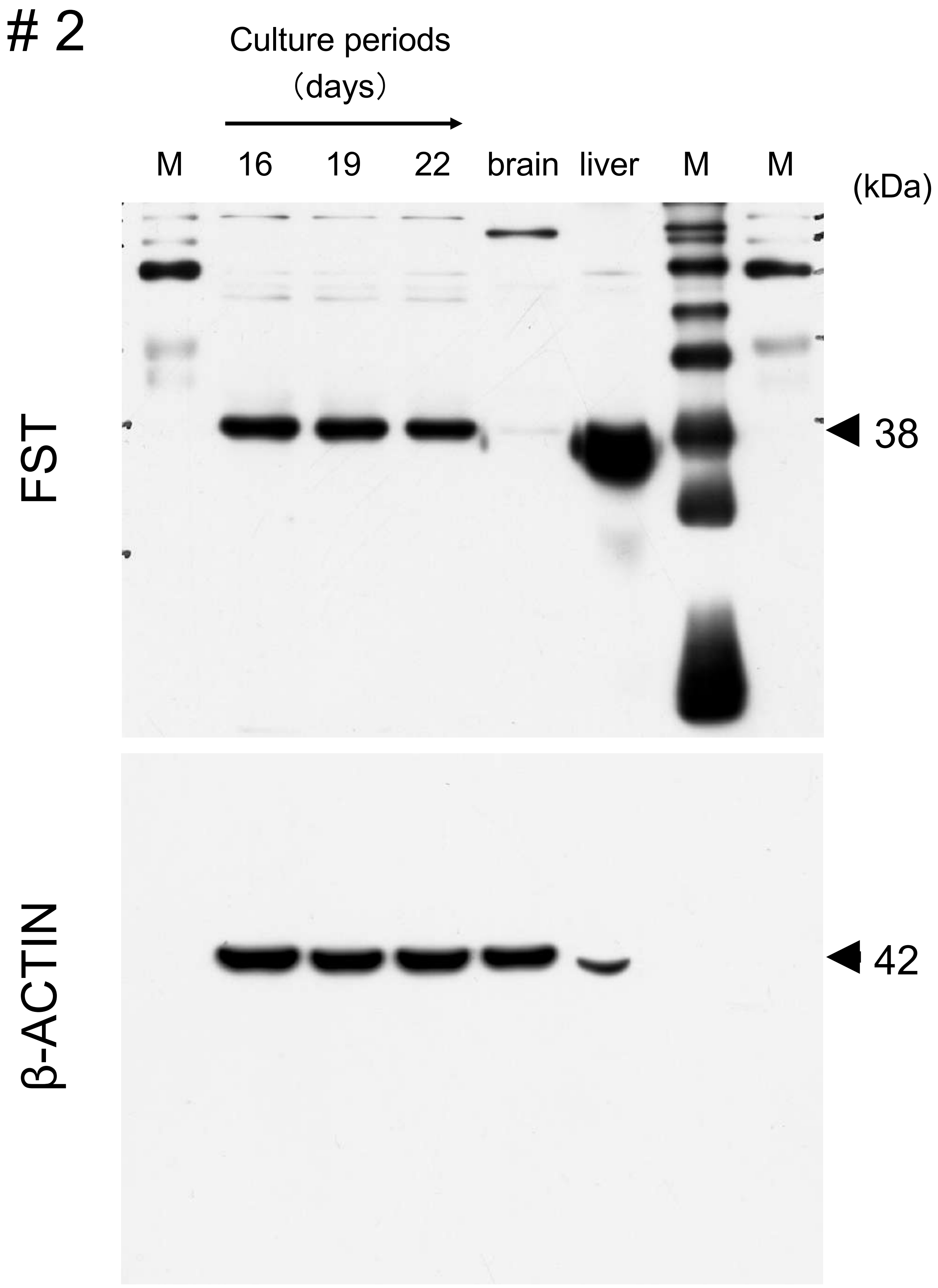

Supplementary Fig. 3

3

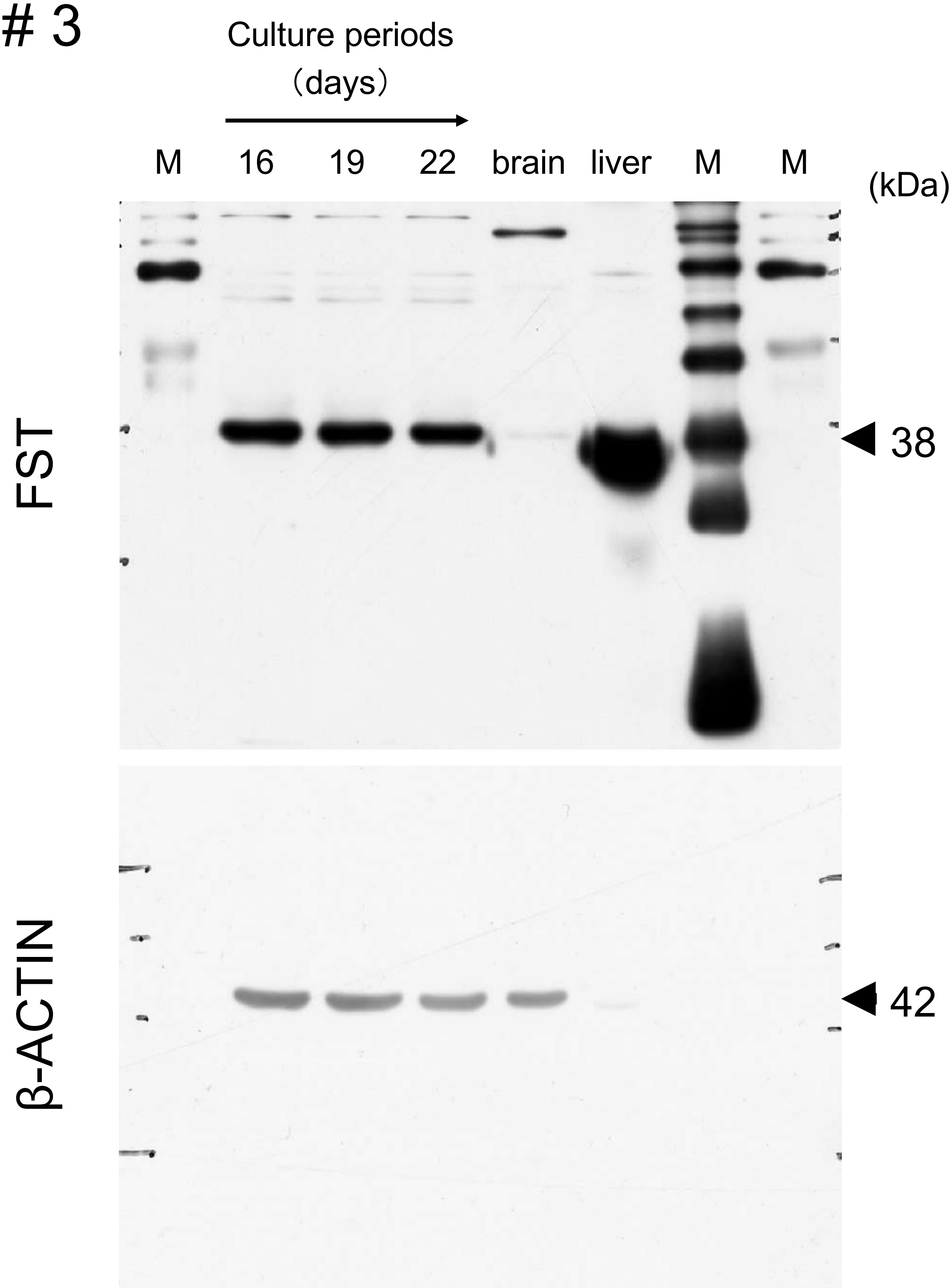

Supplementary Fig. 3

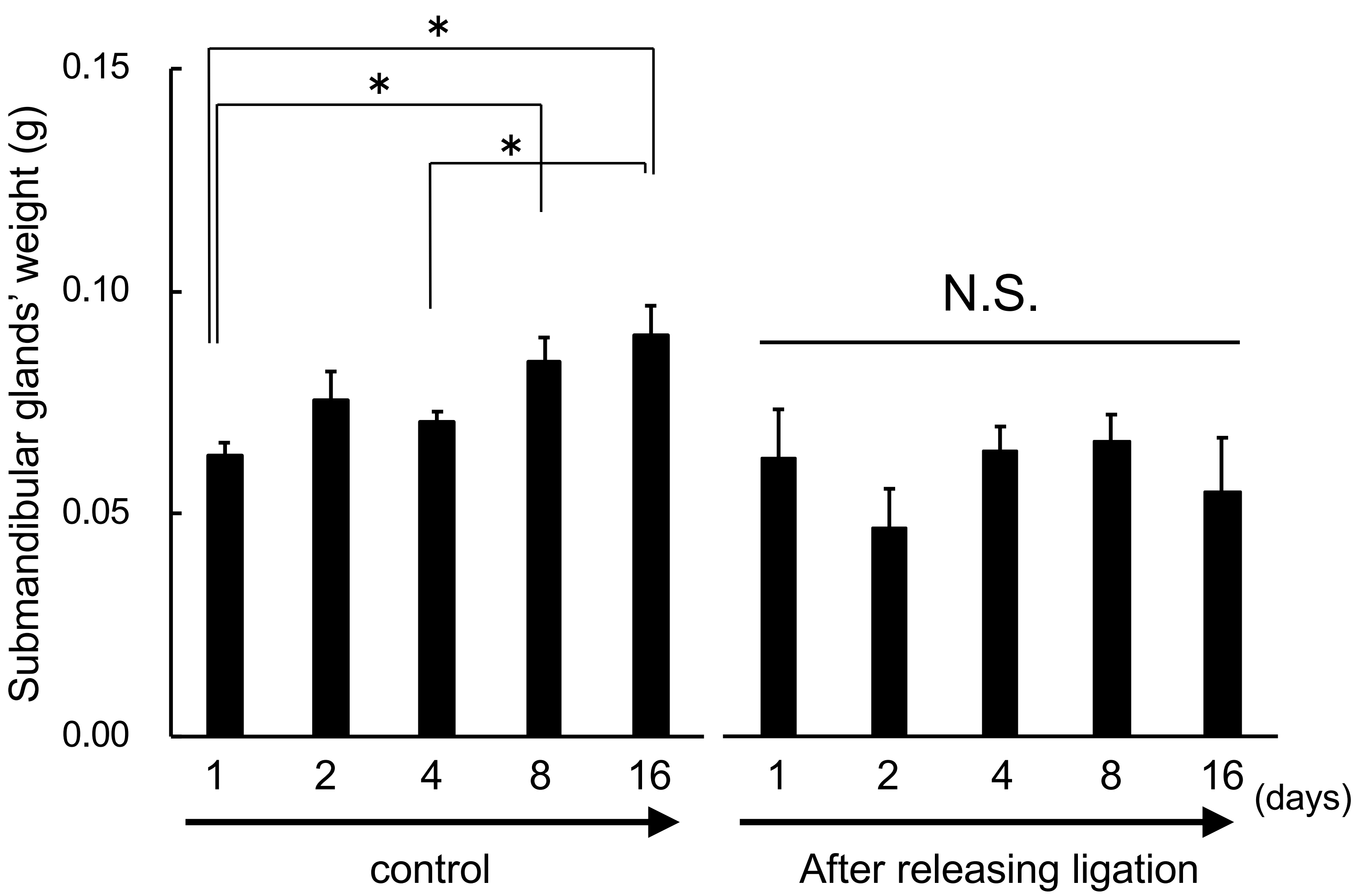

Supplementary Fig. 4

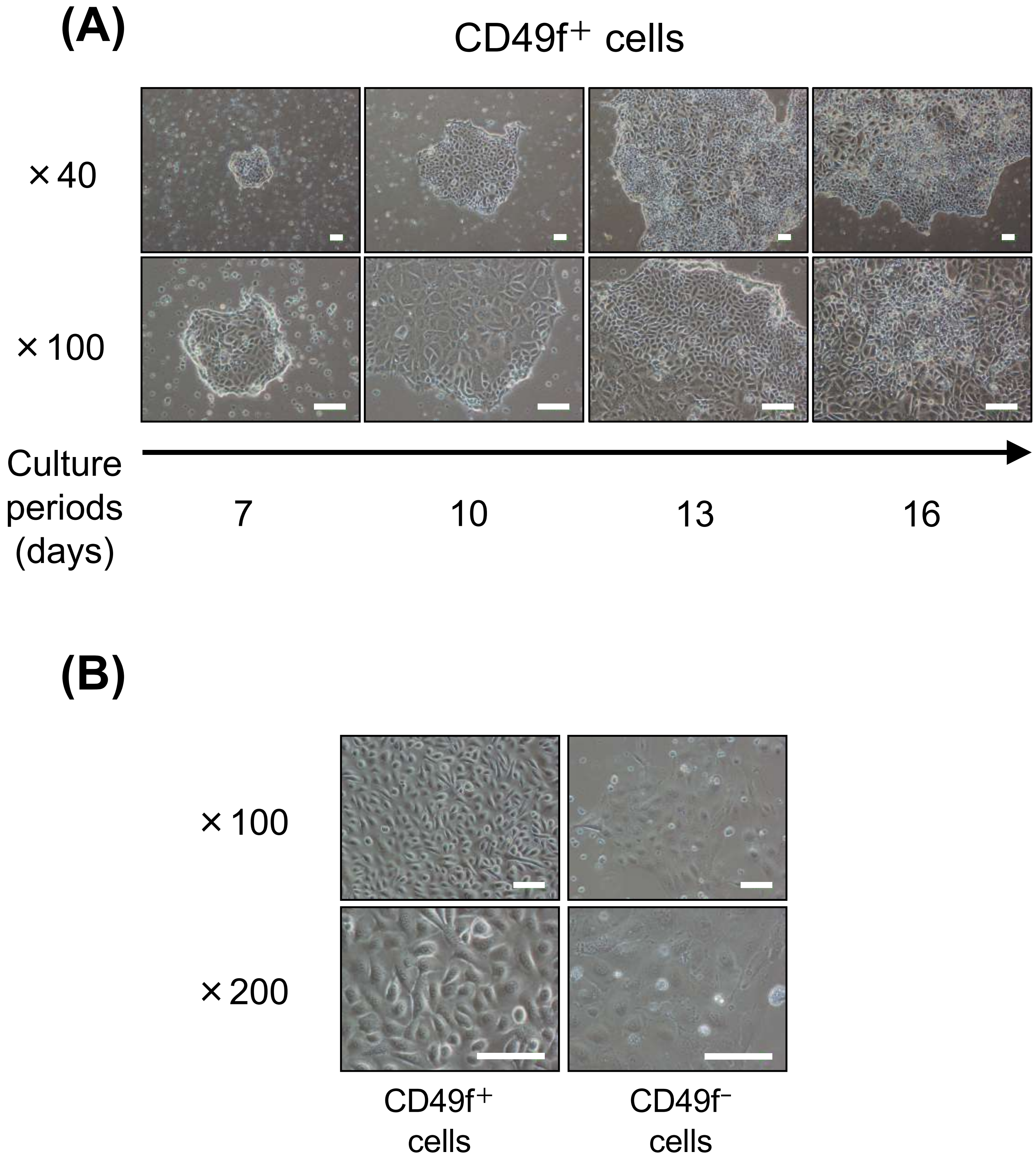

Supplementary Fig. 5

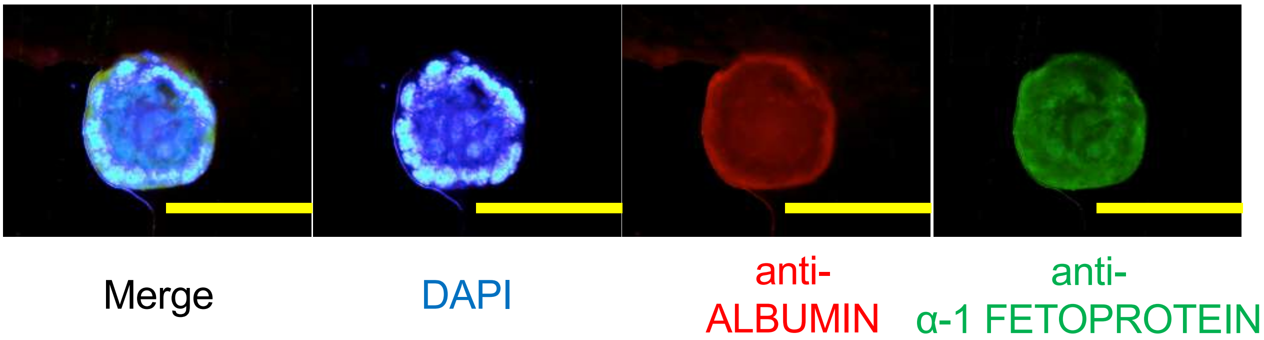

Supplementary Fig. 6

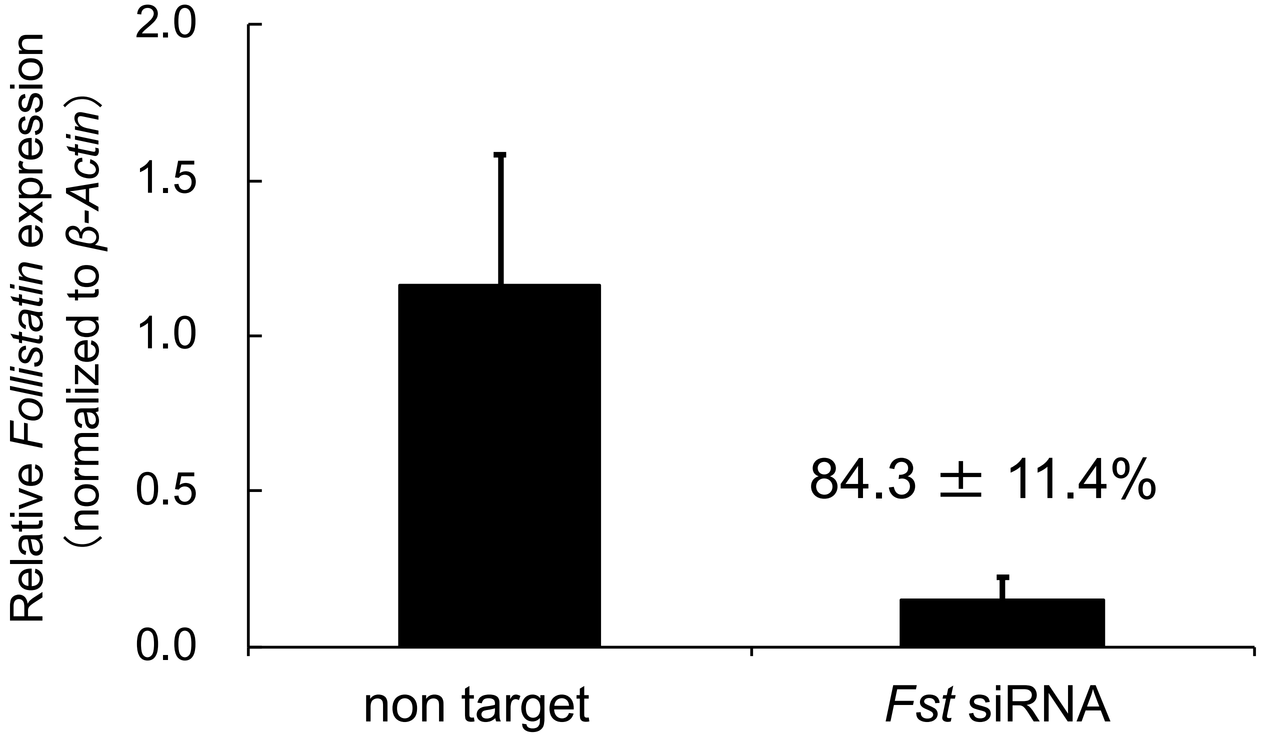

Supplementary Fig. 7

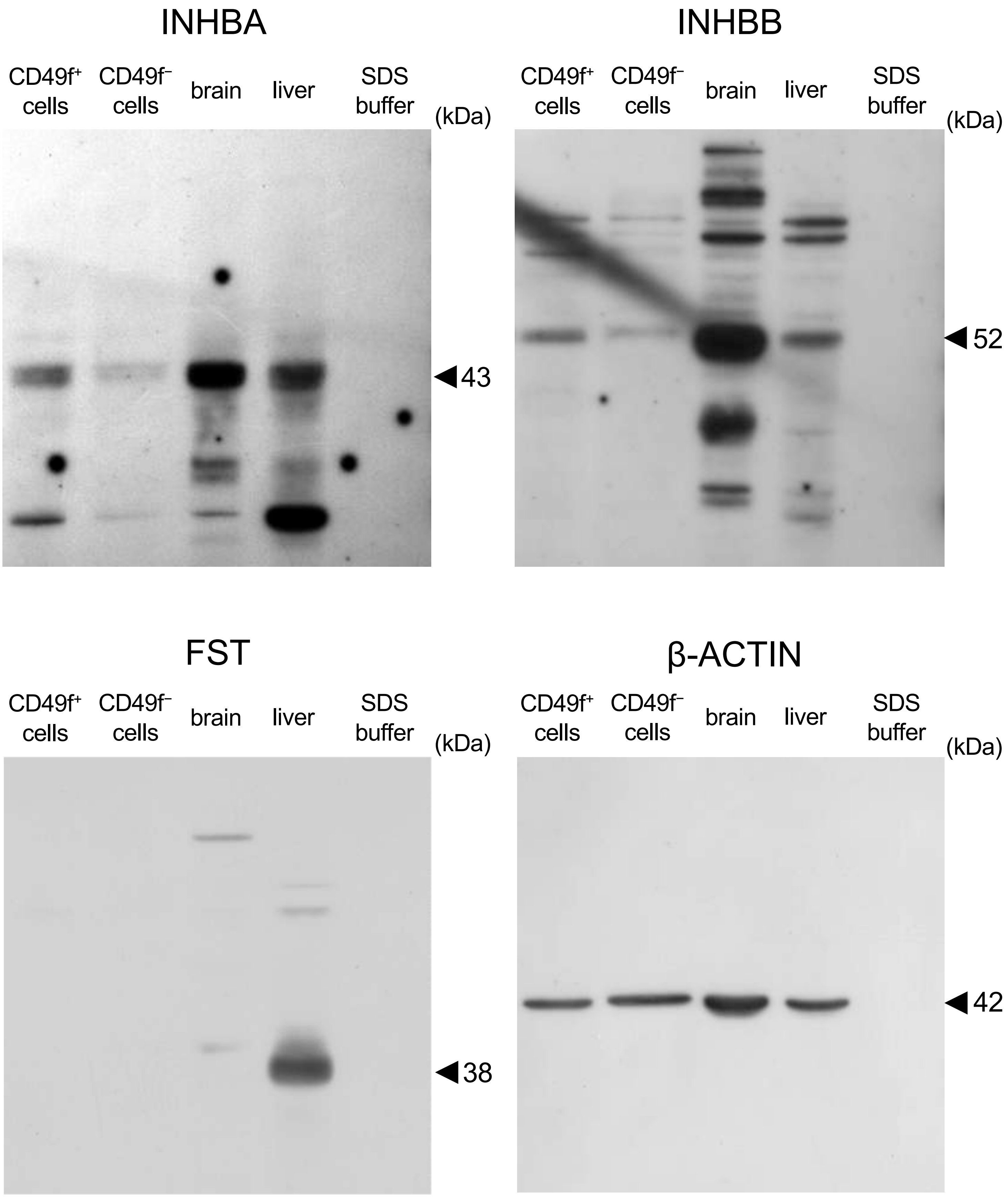

Supplementary Fig. 8

Gene names	Ave. Ct values
β-Actin	14.699
Acvr2a	21.326
Acvr2b	24.685

Supplementary Fig. 9
